# Bronislaw Onuf-Onufrowicz (1863–1928)

**DOI:** 10.1007/s00415-018-8784-0

**Published:** 2018-02-08

**Authors:** Filip Marcinowski

**Affiliations:** Warszawski Uniwersytet Medyczny, Warsaw, Poland

The nucleus of Onuf is a discrete group of motoneurons in the sacral spinal cord involved in micturition, defecation, and muscular contraction during orgasm. Its role has been intensively investigated in the recent years. However, Bronislaw Onuf-Onufrowicz (1863–1928), who was the first to describe this structure over 100 years ago, is almost forgotten.

Bronislaw Kazimir Arthur Onufrowicz was born on July 4, 1863 in Yeniseysk, Russian Empire. He was the second son of Adam Onufrowicz and his wife Maria née Kamionko. His family belonged to the Polish nobility, the father was a physician who also had a small goldmine. In 1869, the whole family went to Germany to provide a good education for the children. They stayed in Breslau for 1 year, then Adam went back to Russia, while Maria with five children went to Switzerland. Bronislaw began his education in Industrieschule in Zurich and pursued his medical studies at the University of Zurich. He and his elder brother Wladislaus (1854–1899) became interested in neurology and neuroanatomy. Auguste Forel (1848–1931), the professor of psychiatry in Zurich between 1879 and 1906, was the supervisor of the theses of both brothers. Bronislaw’s dissertation dealt with the subject of the origin of the vestibulocochlear nerve as investigated using Gudden’s degeneration method [1], while Wladislaus wrote about the frontooccipital fasciculus [2].

Bronislaw graduated in 1884 and the next year he was appointed in Forel’s clinic Burghölzli as a volunteer physician, and from 1888, as an assistant physician. Having worked there for 2 years he spent 8 months in the ophthalmology clinic under Otto Haab (1850–1931). Among his colleagues in Zurich were Constantin von Monakow (1853–1930), also an emigrant from Russia, famous for his neuroanatomical work, Eugen Bleuler (1857–1939), who coined the term schizophrenia, and Adolf Meyer (1856–1950), soon-to-be the most important figure in American psychiatry in the first half of the 20th century.

**Figure:**
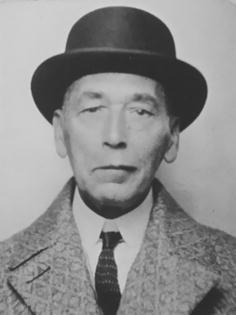
Bronislaw Onuf-Onufrowicz, c. 1920. Courtesy of Prof. Nicholas Onuf

After his training in Burghölzli Bronislaw worked for a short time as a ship physician on Rotterdam-Lloyd steamers in the East Indies and for Holland America Lijn cruise line.

In June 1890, Onufrowicz family came to the USA and settled in Dolgeville, Herkimer County, New York State. Shortly after their arrival, Bronislaw shortened his surname to Onuf; he became an American citizen in 1896. In the USA, Onuf soon built up an extensive practice and continued his scientific work. In 1895 he moved to Brooklyn, where he befriended Bernard Sachs (1858–1944), a renowned New York neurologist. In 1897, Onuf translated Sachs’ pediatric neurology handbook to German [3]. Onuf was appointed to the New York Polyclinic, the Long Island Medical College and served as the consulting neurologist in various medical institutions. From 1894, he has worked as the clinical assistant to the New York Post-Graduate Medical School, where he lectured on electrodiagnostics and electrotherapy. From 1895, Onuf contributed extensively to Smith Ely Jelliffe’s (1866–1945) *Journal of Nervous and Mental Disease*, reviewing foreign books and articles.

Between 1896 and 1901, Onuf was appointed as the associate in the newly established Pathological Institute of the New York State Hospitals, under the direction of pathologist and psychiatrist Ira Van Gieson (1866–1913), where he devoted himself to the study of neuropathology. He was also serving as the consultant in neuropsychiatry to the Ellis Island Immigration Station [4]. Onuf’s diagnostic and language skills (he spoke English, German, Polish and Russian) were probably very useful in this kind of work.

Between 1903 and 1905, Onuf was employed as a resident-pathologist in the Craig Colony for Epileptics in Sonyea, New York State. He investigated changes in the central nervous system and the blood of patients with epilepsy [5].

As early as 1895, Onuf was interested in psychoanalysis. This year, he published a one-page long summary of Sigmund Freud’s paper about defense psychoses. He was among the charter members of the New York Psychoanalytic Society, founded by Abraham Brill (1874–1948) in 1911. Onuf’s work in the field of psychoanalysis included review articles about psychotherapy and interpretation of dreams [6, 7]. When Freud visited New York in 1909, Onuf was sent by Brill to greet him and his companions. In the same year, Onuf was a physician in charge at John F. Louden’s Knickerbocker Hall Sanatorium in Amityville, New York State. This private facility was one of the first in the USA to offer psychoanalysis. In 1915, Onuf opened his own sanatorium for demented patients in Park Ridge, New Jersey, which was later relocated to Rutherford, New Jersey.

Bronislaw Onuf died on December 29, 1928 at his home in Rutherford. He was survived by his wife Alice née Dolan (1886–1967) and twin children. His body was cremated and the ashes dispersed on the family property in Rutherford.

Onuf authored more than 40 original articles from the fields of neuroanatomy, neuropathology, clinical neurology, psychiatry, psychoanalysis and eugenics. The most important works of Onuf were published during his appointment in the New York Pathological Institute. The first one was a monograph written together with Joseph Collins (1866–1950) about the sympathetic system [8] and the second one focused on the spinal cord and described what he called a “nucleus X”, today better known as the Onuf’s nucleus [9, 10]. He was able to determine that this structure is located in the sacral segment of the spinal cord (S1–S3) and he hypothesized that due to its location it contained motoneurons supplying innervation for the ischiocavernosus and bulbocavernosus muscles, playing a role in regulating erection and ejaculation. However, it was not until the 70s, when the works of Mannen et al. showed that motoneurons in the nucleus of Onuf are relatively spared in amyotrophic lateral sclerosis (ALS).
